# “They Could Have Cut My Head Off and I Wouldn’t Have Cared”—A Qualitative Study of Patient Experiences and the Impact of Trigeminal Neuralgia

**DOI:** 10.11607/ofph.3110

**Published:** 2022-12-20

**Authors:** Carolina Venda Nova, Joanna M. Zakrzewska, Richeal Ni Riordain, Sarah R. Baker

**Affiliations:** ^1^University College London, Eastman Dental Institute, London, United Kingdom; ^2^Cork University Dental School and Hospital, Cork, Republic of Ireland; ^3^School of Clinical Dentistry, Sheffield University, Sheffield, United Kingdom

**Keywords:** Patient-centered care, Patient-reported outcomes, Qualitative research, Trigeminal neuralgia

## Abstract

**Aims:** To understand, from the patient perspective, the meaning of living with trigeminal neuralgia (TN) and what the patient-desired outcomes of treatment are. **Methods:** A qualitative study involving focus group work with 14 participants with a diagnosis of TN was conducted. The discussions were recorded and transcribed verbatim and analyzed using framework analysis. **Results:** Four themes and 14 subthemes were identified. Theme 1 reflects the uncertainty about TN etiology and prognosis; theme 2 includes descriptions of the mental, social, and physical impacts of TN that contrast with coping mechanisms developed over time; theme 3 reflects participants’ views of what a successful treatment means and the specific outcomes they expect following treatment, as well as patient willingness to self-manage their conditions while supported; and theme 4 highlights the importance of appropriate and timely access to health care and the importance of peer support. **Conclusion:** This study confirms the need to move beyond the biologic models of disease to patient-centered care and research approaches.

Trigeminal neuralgia (TN) is defined by the International Classification of Orofacial Pain [1] as “a disorder characterized by recurrent unilateral brief electric shock–like pain, abrupt in onset and termination, limited to the distribution of one or more divisions of the trigeminal nerve and triggered by innocuous stimuli.”.

The treatment of TN largely follows the biomedical model, with most studies describing the implementation and/or effectiveness of pharmacologic and/or surgical treatments. Moreover, the outcomes under investigation are typically limited to the effects of these types of treatments on pain intensity levels or the degree of pain relief. Few studies to date have explored—in depth—the experiences of patients from their own perspective utilizing qualitative methods, nor examined the wider psychologic and social impacts of TN.

A recent systematic review of 467 TN intervention studies identified only 46 that reported the effect of TN on physical impact and 17 on emotional impact. Yet it is well established [2, 3] that pain can be influenced by a very wide range of factors [4, 5, 6]. One study reported outcome data of 225 patients and identified that more than 50% were anxious, and a high proportion of patients showed negative thoughts about their pain on the Pain Catastrophizing Scale (PCS; n = 146/188) [6]. Another study looking at the burden of illness of TN identified that patients who completed the PENN Facial Pain Scale-Revised (n = 77/127) indicating pain interference had worse quality of life as reported on the EuroQoL-5 Dimensions-5 Levels (EQ-5D-5L) index when compared to those reporting no pain interference (0.80, SD = 0.214 *vs.* 0.96, SD = 0.141, respectively) [4].

Indeed, the wider pain community has been focused on exploring outcomes of treatment other than pain intensity (*e.g.*, quality of life [QOL], social interactions, sleep, fatigue) and are working with patients to fully understand the extent of disability caused by their chronic pain condition, for example, in the fields of migraine [7, 8], nonspecific lower back pain [9], and complex regional pain syndrome [10].

Patients have seldom been involved in TN studies in a way that would allow the incorporation of constructs that are important to them in prospective clinical studies [2]. Allsop *et al*. [11] conducted a qualitative study with focus groups involving 16 TN patients and identified 4 main themes (diagnosis and support with TN; living in fear of TN pain; isolation and social withdrawal; and medication burden and looking for a cure). The study, however, did not aim to expand current understanding of what patients consider to be meaningful outcomes of treatment. These are essential in order to determine the best treatment approach [12].

The aim of the current study was to (1) capture the description of the lived experience from the patient perspective and (2) to highlight important treatment outcomes to inform an online Delphi survey with different stakeholders with the aim of developing a Trigeminal Neuralgia Core Outcome Set for clinical trials and other prospective studies (https://www. comet-initiative.org/studies/details/1123).

## Materials and Methods

This was a qualitative study incorporating online focus groups with TN patients. The reporting of the study follows guidance from the Standards for Reporting Qualitative Research (SRQR) [13].

### Ethical Considerations

This study received ethical approval from the North of Scotland Research Ethics Committee (19/NS/0153). Those willing to participate were sent the study information leaflet and a consent form via email, which they completed, signed, and returned before their allocated focus group.

### Research Team and Reflexibility

The research team consisted of four researchers (three oral medicine clinicians and a health psychologist). One of the supervising researchers (J.M.Z.) identified potential participants, and the other supervising researchers (R.N.R. and S.R.B.), who have extensive experience in qualitative study designs and focus group work, supervised the focus groups. The lead researcher (C.V.N.) was responsible for the recruitment of participants and running the focus group. Although this researcher was also a clinician, they introduced themselves as a researcher, as the role of the facilitator might impact the behavior of the participants [14]. At the end of each focus group, a debrief meeting was conducted with emphasis on the facilitator’s reflections and self-appraisal on their role as facilitator, researcher, and clinician in order to minimize risk of bias [15], and feedback was provided to the group facilitator by two senior researchers (R.N.R. and S.R.B.). The analysis of the data was done independently by C.V.N. and S.R.B.

### Participants and Sampling Strategy

The sampling strategy aimed to include participants who had different TN phenotypes (*i.e.*, different disease characteristics according to the International Classification of Orofacial Pain [1]) along with participants who had been offered or who had received different treatments and those who had experienced TN for a range of durations.

Participants older than 18 years with a diagnosis of TN who attended National Health Service (NHS) facial pain clinics in London and in Sheffield, from February to August 2020, were asked by their attending clinicians if they were willing to participate in the study. Participants needed to have a good command of the English language and be willing to participate in an online group discussion to be considered for the study. There were no other inclusion or exclusion criteria. Fig. 1 shows the flowchart of participants.

**Fig. 1.** Flowchart showing patient recruitment.

A total of 36 patients were contacted by telephone between August and September 2020. Three focus groups were run using an online platform (Zoom Video Communications) with a total number of 14 participants. Table 1 displays the demographic characteristics of the participants. Due to the COVID-19 pandemic, the focus groups had to be conducted online.

**Table 1.** Demographic Characteristics of Participants.

Total no. of participants		14
Gender, n (%)		
	Female	8 (57)
	Male	6 (43)
Mean ± SD age, y		
	Total	57.4 ± 10.9
TN classification, n (%)		
	Classic	7 (50)
	Idiopathic	7 (50)
	Secondary	0
Mean ± SD disease duration, y		
		7.6 ± 4.8
Current management, n (%)		
	Medication	13 (93)
	In remission	1 (7)
Previous surgery, n (%)		
	Any surgery	5 (36)
	MVD	4 (80)
	Radiofrequency	1 (10)

*MVD = microvascular decompression.*

There are many advantages noted in the literature for conducting focus groups online rather than face to face. These include convenience for the participant to be able to join from their home, participation of a geographically disperse group, and reduction of traveling and related expenses, all of which appear not to compromise the quality of the data obtained [16]. For face-to-face focus groups, some authors, such as Krueger and Casey [17], recommend overrecruiting to avoid running groups with a small number of participants due to last-minute dropouts and to aim for between 6 and 10 participants per group; however, as the online setting presents its own challenges, it was decided to aim for 4 to 5 participants per group for the present study. Some argue that the unit for analysis of focus group work should be the group itself, and therefore the total number of groups—and not the total number of participants—should be considered when talking about sample sizes [18, 19]. In this study, it was decided to stop recruitment once data saturation was achieved instead of aiming for a minimum number of participants or focus groups. There are variations in the definition of data saturation, but for the purposes of this project, it was defined as the moment at which the data collected had enough breadth and depth that it was adequate to answer the research questions [20].

Focus group discussions lasted between 70 and 90 minutes, and participants were offered a small reimbursement for taking part.

### Data Collection

Focus group methodology was chosen to capture not only participant experiences of living with TN, but, more importantly, to allow group interactions and to allow participants to collectively explore and reach consensus as to what the outcomes of TN treatment should be [21, 22]. The main areas to be explored were (1) the lived experience of TN; for example, how participants would explain their condition to others and what the impact of TN was on different aspects of their lives; (2) what it meant for participants to live through a period where they had pain in contrast to ones in which they were pain free; (3) what participants understood to be a successful treatment; and (4) what treatment outcomes participants thought should be used in future TN studies.

A focus group open-question guide was developed by the research team and checked for relevance and accuracy by a health psychologist (not part of the research team) with clinical experience in TN. The guide was based on the TN and chronic pain literature together with expertise from oral medicine colleagues (**Supplementary material 1**). The questions were slightly edited following the first session based on feedback from participants, specifically relating to the question about their understanding of the condition.

While following the question guide, the facilitator allowed conversations to flow between participants, only intervening either for clarification or to ensure that all the participants were given a chance to speak [18, 21].

The focus group discussions were audio recorded and sent for professional transcription on the same day. The transcription was done verbatim.

### Data Analysis

#### Framework method and conceptual framework

A framework method was used for the thematic analysis of the data [23, 24]. TN, chronic pain, and healthrelated QOL (HRQOL) literature, such as the work conducted by Allsop *et al*. [11] and Zakrzewska *et al*. [6], the biopsychosocial model approach to chronic pain treatment [25], and the Wilson and Cleary HRQOL model [26] were the lenses through which the focus group data were analyzed. The chronic pain biopsychosocial model and the Wilson and Cleary HRQOL models are well established and widely used in the chronic pain world. It was anticipated that themes like biologic factors, such as pain intensity or side effects of treatment, psychologic factors, such as depression and anxiety, and social factors, such as social support and social interactions, would be identified within the obtained data.

Although these conceptual frameworks were used to guide the process of identifying initial codes, an inductive approach to data analysis—where the analysis was data driven—was privileged without attempting a rigid fit on the conceptual frameworks [27].

C.V.N. and S.R.B. independently read and coded the transcripts multiple times and held several online meetings to discuss and refine the candidate themes and subthemes. Data analysis was done manually using Microsoft Word. **Supplementary Table 1** shows an example of the note-taking and coding.

## Results

Four themes and 14 subthemes were interpreted from the analysis of the data of the three focus groups. Table 2 outlines the themes, subthemes, and their descriptions. Each theme will be discussed and key aspects described through the use of quotes from participants. Fig. 2 illustrates the intricate relationships between themes and subthemes, which will be elaborated on in the discussion.

**Table 2.** Description of Identified Themes and Subthemes.

Themes	Subthemes	Description
Characteristics of TN	Descriptors of TN Uncertainty about etiology Prognosis—How chronic is chronic TN pain?	Participants’ descriptions of the condition using vivid vocabulary. Participants’ account of having to deal with a condition for which the etiology is not completely understood and that has an uncertain prognosis.
Impact of living with TN	Psychologic impact Functional impact and daily life activities Social impact Cognitive processes	Account of the many impacts of TN on mental, physical, and social wellbeing—impact on activities such as working, eating, applying lipstick, or kissing or hugging relatives—with emphasis on fear of triggering an attack. Description of coping mechanisms developed by participants over time.
Navigating through treatment outcomes	The meaning of a successful treatment—“I would like to get rid of it [pain] completely.” Negotiating side effects Supported self-management The intricacies of normality	Descriptions of expectations with regard to outcomes of treatment, with emphasis on pain levels, side effects of treatment, quality of life, and mental wellbeing. Participant’s descriptions of their willingness to self-manage if well supported. Definition of what a “normal life” or “going back to normal” is.
Access, awareness, and peer support	Streamlined access to health care Health care professionals’ awareness Peer support	Description of importance of early recognition of the disease by health care professionals, which could speed early referrals to appropriate specialized care. Participant’s account of the importance of the shared experience due to the invisibility of pain.

**Fig. 2.** Interconnectedness of themes and subthemes.

The letters refer to the focus group, and the number to the participant. **Supplementary Table 2** shows a more detailed account of themes and subthemes, illustrated by participant quotes.

### Theme 1: Characteristics of TN

Most participants found it easy to describe the meaning and what prior knowledge they had about TN. All participants volunteered information on verbal descriptors of their pain, but the majority expressed uncertainties about the etiology and prognosis of TN.

#### Descriptors of TN

Participants agreed that different individuals might experience TN differently. Some participants used pain descriptors like “stabbing,” “burning,” and “exhaustive.” One participant used a more vivid description of their pain, comparing it to an angry hedgehog:

*“And I think as X said, it will affect different people in different ways, and depending how long you have been experiencing it, then obviously that may give you a whole different range of symptoms.” —FGB1*

*“Mine is a stabbing and then like a burning and then just exhaustion afterwards.” —FGC3*

*“I describe it more like a hedgehog just popping out from the side of my face, really angry and then throwing me down to the floor . . . a hedgehog inside my face.” —FGC2*

#### Uncertainty about etiology

Most participants described TN as a condition that happens without a clear explanation and that no one exerts control over. They described that it is related to changes in the trigeminal nerve that are uncontrollable, and that the symptoms can happen randomly. Two participants went so far as describing that TN could be an issue related to the myelin sheath, and one of them concluded that TN is random and uncontrollable:

*“It is a condition and it’s something that just happens. I don’t think anybody knows why it happens to some and not to others.” —FGA1*

*“. . . as FGA1 said, there is no explanation as to why it happens or whether there is a cure for it.” —FGA2*

*“My understanding is that . . . it is to do with the trigeminal nerve and I understand that the nerve is shooting and sends pain signals to different parts of your face . . . it could be to do with the myelin sheath wearing away and therefore coming in contact with something else . . .” —FGB2*

#### Prognosis: How chronic is chronic TN pain?

The uncertainty around the etiology of TN linked then with similar uncertainties about the prognosis of TN. Most participants were preoccupied with the long-term behavior of TN, its prognosis, and the longevity of the symptoms. They also worried about the lack of a curative treatment. Two participants used the words “fear” to express how they felt about the prognosis of TN:

*“. . . I was actually quite upset when I realized that I couldn’t actually do anything about it and it’s a long-term thing as well, it is not going to go away overnight.” —FGA4*

*“I guess what comes to mind for me I guess is a fear to be honest, that it’s progressive, that it typically or can get worse over time and so knowing how bad it has been or can be I guess an anxiety around it. If it is progressive, what does that mean?” —FGB3*

*“There is a degree of control which I can exert over it but there’s always the fear factor that it’s going to get worse, it’s going to get more serious, and I am going to experience more of what other people experience having listened to stories from other sufferers.” —FGB1*

### Theme 2: Impact of Living with TN

All participants agreed that TN had a huge impact on different aspects of their life, whether psychologic, social, or physical. For the majority of participants, eating was challenging in times of pain and they avoided any possible pain triggers, such as brushing their hair or applying makeup. Participants displayed hypervigilant behavior either in relation to their medication, what they could eat, or even about the weather (*e.g.*, whether it would be windy or cold). This hypervigilant behavior seemed to be associated with fear regarding uncertainty of the pain.

#### Psychologic impact

All participants recognized the impact that pain had on their mood or emotional state:

*“I think it’s quite an emotional thing anyway, this whole business [ . . . ]” —FGA4*

The word “fear,” as mentioned above, was often used to express how they felt about the unpredictability of the disease and of a new attack. It was often associated with the construct of anxiety. Fear and anxiety were not exclusive to pain-related episodes, but participants also reported them when they were pain free, a point on which all participants in the focus groups agreed:

*“(I agree) that fear and anxiety play a big part and not only when you’re pain free but when you’re experiencing pain and you’re worried that it’s going to get worse.” —FGB5*

*“I think after a long period you are bound to get anxious and depressed because it is changing your life, it restricts your life...I think the difference with ours (pain) is that you cannot, it’s unseen, people can’t tell, you know.” —FGC5*

*“If you can think about a time when you were pain free and in remission, how would you describe this period?” —Investigator “Still anxious sadly.” —FGA3*

*“Is there a consensus that there is a gratefulness and a relief when you are in a pain-free period but an underlying anxiety and worry? . . . Almost a fear to do a thing that you think might trigger it. Would that be right?” —Investigator “Yes, definitely.” —All in FGA*

The psychologic constructs of catastrophizing and hypervigilance were associated with episodes of pain but also with pain-free episodes, prognosis of the disease, medication management, and the side effects of treatment:

*“I never stopped carrying and taking my tablets [ . . . ] I have them in the car, I have them in every bag. I have got them absolutely everywhere in every pocket practically and I have little alarms to remind me to take them.” —FGA1*

*“Like X was saying, everywhere around the house there’s spare Primark glasses and pills so I know that they’re there to hand. So while I enjoy it very much I do know that . . . well I don’t know it’s coming again I just assume it will come back and so, I think it’s probably like being an alcoholic, you kind of think you’re always aware that you can’t do certain things because that will make it come back.” —FGA3*

*“It’s exhausting for you.” —FGA1*

*“It is, yeah. Because you’re always thinking about it. There’s not a moment where you’re not thinking about a pain or the pain’s there, oh what is it today, what is it going to be. You’ve got to take your tablets obviously throughout the day and that reminds you that you’ve got to take them because you’ve got the pain and it just invades all your life really.” —FGA4*

#### Functional impact and daily life activities

The impact on activities of daily living, although mostly associated with eating due to the possibility of triggering pain, also had an emotional impact, as participants tended to avoid close and intimate contact. They needed to be alert to their surroundings and avoid, for example, cold or breezy weather, as they felt that this may trigger a pain episode:

*“I actually have to be careful how I eat. So if I eat a sandwich, for instance, and I press down too hard on my lip then that is totally excruciating so when I’m actually out eating I have to be careful how I eat, how I drink as well. [ . . . ] Obviously going outside the cold and the wind I have to be careful where I go, but especially in winter for me.” —FGC1*

*“Yeah, I’d like to say I completely agree. I find before I go out now I have to check the weather and see if it’s going to be cold or raining and things like that.” —FGC4*

*“I’ve not been able to eat solids for over 2 years, I had to blend all my food so I’m always really anxious people are going to say oh, shall we go for a bite to eat or something like that and then I have to explain why I can’t.” —FGC3*

*“Lipstick is not something that you’d put on. Or I think one of the saddest things I think was just kissing someone.” —FGA3*

*“Oh that is the worst!” —FGA2*

*“Or hugging someone.” —FGA3*

#### Social impact

The social impact of pain was linked to work, social interactions, and participation in social activities, such as dinner parties. Pain in some instances threatened social participation, as participants tended to display avoidance behaviors as self-protection mechanisms in fear of triggering a painful attack. One participant described this behavior as “withdrawal”:

*“I think the word I’d use to describe where I was at is withdrawal. No semblance of normality, you’re very conscious of the potential of what could go on around you and it’s almost like playing a game of statues, you just want to minimize any possible interaction that could cause you kind of movement in your face, talking, eating, whatever it happens to be and I’m under no illusion it makes it very difficult for someone who is living with you just because you become more and more withdrawn.” —FGB4*

A few participants described that TN impacted their work, not only due to the difficulties in talking and communicating effectively with others, but also because they felt the need to hide their disability from others:

*“I only work part time, I work with small children at a preschool so I just push through and try to avoid things if I can. If I can’t speak, I try to avoid talking to people and just keep out of the way.” —FGA4*

*“So I have had TN for 8 years and I did have to take time off work because . . . my job involves talking to a lot of people . . . you kind of start talking and then you think you can’t say what you are diagnosing . . . you look like you don’t know your job.” —FGA3*

*“From a professional capacity [ . . . ] sometimes the symptoms caused difficulties talking. I can work from home so that sometimes reduces the almost embarrassment [ . . . ] because it is not something you actually want to be broadcasting to your colleagues because it becomes a bit debilitating, and you want to keep that from them as long as you can.” —FGB1*

It was striking that participants described feeling isolated as no one could see their pain due to an absence of visible symptoms/signs. The lack of social validation and the seeming lack of knowledge as to how much disability TN could cause resulted in many participants reporting a sense of isolation:

*“My work colleagues know that I have got something wrong, but they don’t fully understand what it is, and I think that is the thing, a lot of people don’t actually understand what it involves and how it affects you because you look normal, we all look as if there is nothing wrong with us . . . so there’s nothing obvious on the outside but they just don’t realize the pain and how it invades yourself when you have it. So, I find that difficult sometimes.” —FGA4*

*“A lot of people think there is nothing wrong with you because they can’t actually see anything at all but they don’t realize how much pain you can be in.” —FGC1*

*“It does not seem that common [TN] and so therefore, other people don’t have any understanding of it [ . . . ] so when it does strike you can feel a little isolated with it. . .” —FGC2*

#### Cognitive processes

Although most participants described changing their behavior to avoid situations where their pain could be triggered, they nevertheless also reported developed coping and adaptive strategies, either through reading, with the help of health care professionals, and a few, on their own, for example, through mindfulness:

*“I think initially I used to think well people used to say well you just need to calm down and I used to think [laughs] I don’t really know that I’m not calmed down and then obviously the more that you read about it or when you actually know that’s what you’ve got it’s easier to accept it and understand that it’s not something that you’re doing, it just happens and the best way then is just to move forward with it.” —FGA3*

*“I think I am in a good place at the moment, but without the support of the clinic, I would feel panic.” —FGB2*

*“But I think I’ve reached the point where I can accept it’s there and I don’t want to take any more medication but if it then starts getting worse then I will increase it.” —FGA2*

*“Mindfulness actually helped me control the pain. It didn’t make it go away but it helped to control the pain and put me back in control.” —FGB1*

Some participants compared TN to other conditions; this shift in their conceptualization of the disease appeared to allow for improved coping mechanisms:

*“When COVID all kicked off and you’re seeing all these poor people being put into intensive care I just thought as long as I’ve got my oxcarbazepine I can cope with anything.” —FGA4*

*“I think sometimes though, if you thought you had heart disease or diabetes you wouldn’t think twice about having to take medication to prolong your life or make you feel better [ . . . ] so if it makes you feel better [the medication] it’s better to increase it [ . . . ] so that you tip over the edge of being in constant pain.” —FGA2*

### Theme 3: Navigating Through Treatment Outcomes

The meaning of a successful treatment was “I would like to get rid of it [pain] completely.” All participants were very clear about their expectation with regard to treatments offered; they wanted to be 100% pain free. Nevertheless, most were willing to compromise having side effects in favor of pain relief:

*“I would have sold my soul to the devil just to change that [the levels of pain regardless of side effects].” —FGC1*

Others would accept having the pain as long as it was less intense and the attacks were less frequent:

*“Yeah, I would say ideally success would be pain free, off the medication.” —FGA2*

*“I think it is a sliding scale actually because anyone who has been lucky enough to get remission from either medication or indeed surgery, the bar is pretty high, so you start saying well, the first thing I’d really expect or like is to get absolute total remission for good.” —FGB4*

*“I think the severity of the pain. If it wasn’t so severe. If I had to have it all the time but it was just dull I would rather that than the sharpness of it. So probably just to reduce the intensity of it.” —FGA3*

*“Yeah, I would like to have something that would just take it away completely— definitely. Those little niggly pains and jumping to big pains and all of that—I’d like to get rid of it completely.” —FGC4*

*“Yeah, I think the intensity as well. I can live with it if it’s just there. I can live with that but when it gets to be really intense that’s the thing for me, definitely. It’s just getting rid of that intensity of pain, definitely . . . Like if you have this constant dull ache and it just stays there that’s fine, it’s when you have the dull ache and then it’s going [makes shooting noises].” —FGA4*

*“Yeah, that’s the worst thing. Yes, the shootingness that just catches you. Once you’ve got that under control you kind of think oh, actually I’m so much better than I was.”—FGA1*

#### Negotiating side effects

A few participants worried about side effects of surgery, specifically numbness, and would not consider surgery because of this, unless it was their last option. Most participants had experienced medication side effects, specifically related to weight gain and feeling slower, drowsy, and forgetful. Most participants appeared to accept some side effects providing their pain was well controlled:

*“I would say it would be nice not to have the side effects, but if you balance that against the pain, I would rather have the side effects than the pain.” —FGB2*

*“I would agree with that entirely. I find myself now and again, [ . . . ] searching for the right word. Definitely tired. [ . . . ] It could be controlled for me and absolutely I would take the side effects in a heartbeat over the condition.” —FGB3*

*“I just think the fear of numbness as well, with the surgery I don’t know if I’ll be able to live with being numb rather than in pain. I’d rather have this dull ache than have a numb face I think.” —FGA3*

*“So, if you had to choose the outcomes of treatment, would these be pain relief, intensity, looking at the quality of life you have living with TN and the side effects of medication?” —Investigator*

*“Yes!” —All in FCG*

#### Supported self-management

Most participants agreed that having support with their mood and with coping strategies would be beneficial, specifically as their coping mechanisms could be influenced by different stages of their journey, whether they were in remission or not. For example:

*“Ideally pain free either with the drugs or into remission [ . . . ]—that would be fantastic. But I also think on top of medication it’s being able to have support through talking therapies and when I was in a really bad state, I did talk to a clinical nurse specialist and that really helped . . . although she couldn’t take away the pain it just helped me. So I’d come off the phone feeling right, I can do this, I can do this so it was really helpful. I wouldn’t take her away, she’s a really important part of it.” —FGB5*

*“So I think ideally complete pain relief but if not then at a level that I feel that I could cope with it.” —FGA1*

*“For me it depends on what particular point you are at. If you are in a period of remission you just want it to carry on and you’d like a magic pill for it never to happen again [. . .] when you are in the darkest days I could find myself saying, if this could reduce to this particular levels I could accept it.”—FGA2*

*“I would agree that fear and anxiety play a big part and not only when you are pain free but also when you are experiencing pain and you are worried that is going to get worse. So in terms of treatment, I would say that for me, it would be really nice to be able to talk to somebody, a professional really regularly to just talk through things. I think that’s really important because sometimes you do feel on your own. [. . .] maybe a psychologist or somebody you can just talk things through with and I think the very action of actually talking about it would really help.”—FGB5*

#### The intricacies of normality

Finally, some participants gave an account of what normal or almost normal life would look like to them:

*“So about 6 months ago I was absolutely just pain free, no symptoms, back to normal.” —FGA2*

*“So it’s never quite back to normal because I’ve got that in the back of my mind, little things that I know might be triggers, even though I’m having a period when I haven’t got the pain I’m just very, very careful eating different things and stuff just to keep it off, keep it away just in case.” —FGB5*

*“I think it’s being pain free definitely, that’s the main thing because that’s the main thing that affects everything isn’t it really, is having that pain and if you haven’t got the pain obviously you can just carry on your life as normal. So I think the pain side of it is for me the most important, definitely.” —FGA4*

### Theme 4: Health Care Access, Awareness, and Peer Support

#### Streamlined access to health care

All participants agreed that having streamlined access to health care support was extremely important. One participant compared losing the support of her consultant to losing her own mother:

*“So even when I saw her in March she sort of said do you think you still need me? And it’s like losing your mother, it’s like oh God, yes do not suggest that I don’t come anymore because I know I don’t have it right now but I really don’t want to be back there to the point of having to go to see her more regularly.” —FGA3*

*“I would like to have someone at the end of the phone I could speak to, if I have got any problems. I have spoken to the clinical nurse a few times and she helped me with medication. [ . . . ] So it is always nice to have someone there just so they can understand what you are going through and just to help you out with things.” —FGC4*

*“I felt it was really through Dr Y, she said try to get more sleep, try to exercise a bit, try to get that balance right which luckily with my husband we arranged that or sorted it so that I didn’t work full days for a while and did that sort of thing. So, since last October through going to see Dr Y, I’ve reduced my medication and at this moment I’m not taking any. So while you all, like X said, look forward to a life of constant medication there is I think a light.” —FGA3*

#### Health care professional’s awareness of TN

Most participants agreed that health care professionals, mainly general practitioners (GPs) and dentists, should be aware of TN, not only to support patients through their journey but to avoid delays in care or unnecessary treatments:

*“X had a really good experience with a GP but generally, GPs don’t understand that it’s such a rare condition they don’t understand what it is and they don’t understand that your medication can change, that you can be on something and then you need to reduce it and then you need to come up again or you need to change to a different drug.”—FGB4*

*“I think it’s upsetting that the dentists don’t realize that TN could be the reason for your pain. Why don’t the dentists know in the first place to explore that option? Because I thought mine was dental as well, to start off with [ . . . ] why don’t the dentists consider that as an option before they start taking nerves out and messing about with your teeth? I find that really upsetting and annoying because I think the dentists should be more aware, at the end of the day.” —FGA4*

*“It took so long to get diagnosed that it just adds to the misery of it.” —FGC3*

#### Peer support

Participants also felt that talking to others with the same disease could be helpful and offer hope:

*“I think it’s nice just to speak to other people. I’ve never actually spoken to anyone else that has got this apart from the people that I’m speaking to today. Just to speak to people and you know, like X said, today she is pain free and she has been for a while, I think just that reassurance that it can happen and obviously you know that yourself. But then I think just to speak to other people and to get an understanding from other people from the things that they’ve been through I think is really helpful. And everyone has that fear and you have to deal with the fear. It’s really nice to speak to other people.” —FGA4*

## Discussion

This is the first qualitative study to examine the preferences of TN patients regarding outcomes of treatment. Participants clearly defined pain reduction as the most important treatment outcome, although improving QOL and having supported selfmanagement were also important. This study provides a comprehensive and detailed account of the meaning of living with TN. Like so many other chronic pain conditions [28, 29, 30], TN has impacts on different aspects of daily living.

Although 4 themes and 14 subthemes were constructed from analyzing the data, these are not to be interpreted individually given the underlying interconnectedness between them (Fig. 2). The pain attacks’ unpredictable nature and the long-term prognosis of the condition (theme 1) have an impact on the participants’ mood (theme 2). Due to the same unpredictability (theme 1), participants have avoided attending social events in fear of an attack (theme 2), which in turn has caused upset (theme 2). The subthemes included in theme 2 are strongly interrelated. The participants’ accounts of their actions in social circumstances, whereby they actively change their behavior to avoid triggering pain by avoiding eating in public or by not kissing or hugging others, are indicative of a coping strategy—albeit perhaps a maladaptive one—due to the negative impact on their emotions. Positive examples contrast with the latter, whereby participants have tried to develop ways of managing their pain experience by using alternative treatments, reading about and improving their knowledge of the condition (theme 1), or even by trying to manage their medication intake to minimize the intensity of their pain (theme 3). Similarly, participants’ reappraisal of their long-term condition in comparison to other chronic conditions (theme 2) allows for a reconceptualization of TN and improved coping mechanisms.

Unsurprisingly, the conceptual frameworks used as the initial guide provided accurate constructs, helping to make sense of the gathered data. The biologic pain-related constructs, such as pain characteristics, feature in the biopsychosocial model of chronic pain [34] and in the Wilson and Cleary HRQOL model [26]. These are not static models, and these constructs have a dynamic relationship with subsequent ones, as the level of pain might influence one’s ability to eat or the unpredictability of the disease may influence someone’s fear of an attack, which in turn can cause behavioral changes. The qualitative work by Allsop *et al*. [11] showed the importance of fear in the journey of participants living with TN. Similar to the experiences described in their work, participants in the present study used the word “fear” very often and linked it to many constructs, such as prognosis (theme 1), anxiety and behavioral changes (theme 2), the side effects of treatment (theme 3), and in peer/health care support (theme 4). The risk of pain-related disability caused by avoidance behaviors is well known in the field of pain psychology [2]. Participants’ descriptions of how their behavior needs adjusting due to fear of pain are consistent with the fear avoidance model (FAM) [31], which is often used as an alternative to the biopsychosocial model [32]. The FAM illustrates how, through learning, behaviors change to prevent painrelated stimulus. The behavior is then maintained, and it is argued that this contributes not only to the maintenance of pain-related fear [31] but also to increased pain [33]. There is a dynamic and possibly reciprocal association between constructs, although this reciprocal relationship is yet to be confirmed in TN patients. Constructs of fear and behavior are modifiable [32] and might be used in the multifaceted treatment of TN.

Participants’ descriptions can also be interpreted using Leventhal’s common-sense model of selfregulation [34]. This is another dynamic framework that outlines the emotional and cognitive process that patients go through when faced with a threat to their health. For example, the unpredictable characteristics of TN (theme 1: cognitive illness representation) can impact on emotional wellbeing (theme 2: emotional illness representation). Cognitive and emotional illness representation drive cognitive processes of coping (theme 2). This process is illustrated by the behavior of participants who kept their medication accessible in different areas around the house in case of an attack.

Health care systems and communication about health and disease can influence illness representation [34]. In this group, participants did emphasize the importance of an accurate and timely diagnosis and of support from health care providers (theme 4). Although not modifiable through treatment, changes to the way information is transmitted to patients and the improved accessibility to services are likely to contribute to the patient’s journey, decreasing the emotional distress caused by uncertainty of diagnosis and prognosis (themes 1, 2, and 4). Participant descriptions have highlighted their ability to shift their response to disease and its management (subtheme 7). This is specifically achieved by using mechanisms of social comparison to those suffering from diabetes and heart disease and their requirement for long-term medication, as well as by stratifying and reordering goals, for example, in comparison to those suffering from COVID-19. These examples are better explained by the theoretical model of response shift, which describes a shift in response to a person’s appraisal of a construct; for example, perceived QOL, either by a change in internal standards, values, or conceptualization. The change would be driven by behavioral, cognitive, or affective mechanisms influenced by the person’s antecedents, which can impact on how they appraise the construct under study [35]. This model has recently been revised to allow a distinction between the construct and the measurement of the construct by using, for example, a patient-reported outcome, as well as to allow a response shift to be investigated at different time points [36]. This is important for TN patients, as their understanding of different constructs will likely change depending on if they are in pain or pain free and should be taken into consideration when analyzing patient-related outcome data.

When asked specifically about what outcomes they wished to have following treatment, participants clearly agreed on complete pain relief or reduction in pain intensity as the primary goal of treatment, but they also agreed that side effects and improving QOL were relevant. These findings are not surprising, and a recent European survey of 487 chronic pain patients identified that their main goals were pain reduction (91.18%), taking part in family and social activities (72.55%), and household tasks (68.14%) [37]. Although reducing pain is of utmost importance, as demonstrated by how participants drew a parallel between “normal life” and a “life without pain or pain free” (subtheme 11), having support to engage in self-management, either with the emotional impact of TN or with their coping mechanisms, is important (subtheme 10), and, as discussed above, constructs such as anxiety, fear, avoidance, and coping arose from the participant’s descriptions of their journeys. A recent study of a six-session cognitive behavioral therapy (CBT) group program for TN patients highlighted its potential benefit in reducing the negative beliefs about pain, and participants felt more confident in self-managing their symptoms [38]. Although this was a preliminary feasibility study done with 15 patients, the high levels of patient satisfaction at the end should encourage further studies on psychologic interventions in the management of TN.

## Limitations

Participants with secondary TN—those with TN caused by multiple sclerosis or a tumor—were not recruited. Although the present authors anticipate that their pain experience is similar and their opinions about outcomes of treatment the same, their preferences have not been able to be noted. Some patients declined participation due to the fact that the study had to be carried out online, and this may have acted to bias the sample toward those in particular socioeconomic groups/younger populations. The internet use uptake, although having gone through an overall increase over the last decade, is still mostly seen in younger generations according to the Organization for Economic Co-operation and Development (OECD; in 2016, over 95% of 16- to 24-year-olds in the OECD *vs.* 63% of 65- to 74-year-olds used the internet) [39].

Finally, this study was conducted in the UK, where participants have access to free health care within the NHS. This will be varied around the globe, and therefore the experiences of TN patients might be different from those in the UK.

## Conclusions

These data have important implications for the field of TN. For the first time, patient views were considered, and we have identified outcomes that matter to patients. Through their direct accounts, and by analyzing and interpreting the data, it was possible to appreciate the intricacies and complexities of living with a chronic and unpredictable painful condition. More importantly, as so many dimensions of the condition have been highlighted, there is an urgent need for clinicians and researchers working in the TN field to make a definitive move away from the biologic model toward a multidisciplinary approach that adopts a biopsychosocial model of TN.

## Highlights/Clinical Research

• Resolution of pain is the priority in TN treatment, but improvement of QOL and psychologic support are also important to patients.

• Themes and subthemes identified can be used for the future development and exploration of a TN-specific conceptual framework.

• Themes and subthemes identified might act as modifiers or moderators of disease outcomes and are possible targets for intervention.
